# Integrating stochasticity and network structure into an epidemic model

**DOI:** 10.1098/rsif.2008.0410

**Published:** 2008-10-30

**Authors:** C. E. Dangerfield, J. V. Ross, M. J. Keeling

**Affiliations:** 1 Mathematics Institute, University of Warwick, Gibbet Hill Road, Coventry CV4 7AL, UK; 2 King's College, University of Cambridge, Cambridge CB2 1ST, UK

**Keywords:** noise, networks, pairwise moment closure, diffusion approximation

## Abstract

While the foundations of modern epidemiology are based upon deterministic models with homogeneous mixing, it is being increasingly realized that both spatial structure and stochasticity play major roles in shaping epidemic dynamics. The integration of these two confounding elements is generally ascertained through numerical simulation. Here, for the first time, we develop a more rigorous analytical understanding based on pairwise approximations to incorporate localized spatial structure and diffusion approximations to capture the impact of stochasticity. Our results allow us to quantify, analytically, the impact of network structure on the variability of an epidemic. Using the susceptible–infectious–susceptible framework for the infection dynamics, the pairwise stochastic model is compared with the stochastic homogeneous-mixing (mean-field) model—although to enable a fair comparison the homogeneous-mixing parameters are scaled to give agreement with the pairwise dynamics. At equilibrium, we show that the pairwise model always displays greater variation about the mean, although the differences are generally small unless the prevalence of infection is low. By contrast, during the early epidemic growth phase when the level of infection is increasing exponentially, the pairwise model generally shows less variation.

## 1. Introduction

From the early ordinary differential equation (ODE) compartmental models (Anderson & May 1983) to more recent spatially structured stochastic simulations (Riley 2007), models of infectious diseases have impacted on both our understanding of epidemic spread and public health planning. However, one of the key issues in using such models is a comprehensive understanding of when complexity is important and when it is a superfluous detail. Here, we offer an analytical insight into the confounding roles of stochasticity and spatial structure in the dynamics of infection.

The chance nature of epidemiological processes can have a profound impact on disease dynamics, leading to a range of phenomena not predicted by deterministic models. Standard models based on ODEs are essentially clock work, predicting an identical outcome from the same initial conditions—in addition, such deterministic models generally predict constant endemic levels of infection in the long term (Anderson & May 1992; Keeling & Rohani 2007). By contrast, by capturing the chance nature of transmission and recovery, stochastic models predict variability in the levels of infection. Such variability has two substantial consequences. Firstly, stochastic epidemics are prone to extinction either immediately following invasion (Bartlett 1956) or even once established (Bartlett 1957; Bolker & Grenfell 1995). Secondly, the interaction of stochasticity with the natural oscillatory behaviour of epidemics can lead to large-scale oscillations—termed stochastic resonance (Dushoff *et al*. 2004; Alonso *et al*. 2006). The most common method of studying stochastic models is through simulations using techniques such as the Gillespie algorithm (Gillespie 1977); however, more recently analytical techniques such as moment closure approximations (Keeling 2000*b*; Nåsell 2003), diffusion approximations (Clancy *et al*. 2001; Ross 2006) and exact Kolmogorov forward equations (Keeling & Ross 2008) have all been used to understand stochastic epidemic dynamics.

Spatial structure impacts on epidemic dynamics at a variety of scales. Humans generally live in relatively large communities (e.g. towns and cities) and this compartmentalization often induces spatial heterogeneities, with strong transmission of infection within communities but weaker transmission between communities, capturing the general assumption that transmission strength decreases with separation (Xia *et al*. 2004; Riley 2007). This large-scale heterogeneity has been captured in a wide number of contexts with both spatial (Gibson & Austin 1996; Keeling *et al*. 2001; Mangen *et al*. 2002; Ferguson *et al*. 2005) and metapopulation models (Bolker & Grenfell 1995; Finkenstädt & Grenfell 1998; Smith *et al*. 2002; Hanski & Gaggiotti 2004). However, at a more local scale the dynamics of infection are strongly influenced by the network of available contacts through which infection can be transmitted. The use of network-based models to simulate the local spread of infection through social or sexual contacts has seen substantial advances over the past decade (Watts & Strogatz 1998; Potterat *et al*. 1999; Klovdahl 2001; Halloran *et al*. 2002; Keeling & Eames 2005; Boccaletti *et al*. 2006). In general networks have three main influences over the epidemiological dynamics. Firstly, local connectedness and clustering leads to strong negative spatial correlations between infected and susceptible individuals rapidly leading to reduced transmission, reduced growth rate and therefore higher numbers of susceptibles at equilibrium (Keeling 2005). Secondly, heterogeneities in the number of contacts automatically generate high- and low-risk individuals and hence heterogeneities in the distribution of infection (Eames & Keeling 2002; Keeling & Eames 2005). Finally, the long path length that may exist between individuals (Watts & Strogatz 1998) can lead to asynchronous behaviour across the network (Boots & Sasaki 1999). These three influences shape how individual-level epidemiological characteristics translate into population-level behaviour and hence are vitally important for predicting the impact of control measures, such as contact tracing, that are focused towards the individual (Eames 2007).

In this paper, we focus on bringing together two highly successful approximations—the diffusion approximation for stochastic populations and the pairwise approximation for dynamics on a network—in order to understand the interaction between local spatial structure and stochasticity. In particular, we consider a susceptible–infectious–susceptible (SIS)-type model, which is a good description of many sexually transmitted infections (Anderson & May 1992; Andersson & Britton 2000; Keeling & Rohani 2007) and host–parasite diseases (Stone *et al*. 2008). Despite its applicability, the SIS model provides one of the simplest descriptions of disease transmission as individuals can be in only one of two states: susceptible or infectious.

We initially outline some relatively standard results: the dynamics of the deterministic (homogeneous-mixing or mean-field) SIS model (Anderson & May 1992); the Ornstein–Uhlenbeck (OU) diffusion approximation (Kurtz 1971) to the stochastic SIS model; and the deterministic pairwise equations for SIS-type infections spreading through a network (Eames & Keeling 2002). These results and the associated methodologies will then be combined to create a diffusion approximation to the stochastic pairwise SIS model and ultimately to calculate the associated variances and covariances. These results will be compared with those of two other stochastic models: the stochastic homogeneous-mixing SIS model and the full stochastic Monte Carlo (continuous-time Markov chain) network simulation.

## 2. Basic methodology

While the results and methodologies given below are described in far more detail in other publications, here we briefly summarize the techniques and salient results to more naturally lead the reader into the complexities that follow.

### 2.1 The deterministic SIS model

The simple SIS equation is one of the foundations of predictive models of sexually transmitted infections (Anderson & May 1992). Treating *S* and *I* as the *proportion* of susceptible and infectious individuals in the population, and ignoring birth and death, the SIS equations are2.1
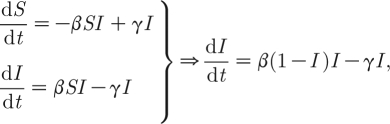

where the single equation is derived from the fact that *S*+*I*=1. We note that if we wish to deal with *numbers* of susceptible and infectious individuals within the population then for consistency the transmission rate *β* is divided by the total population size *N*; *γ* is the rate at which individuals recover and move from the infectious to susceptible state. We refer to equation (2.1) as the deterministic mean-field model. The ability to switch between proportions and numbers will be vital when we consider integer-based stochastic models and their diffusion approximations. For the standard deterministic model it is easy to show that the non-trivial equilibrium point is 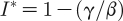, which is stable (and the disease-free state is unstable) if the basic reproductive ratio 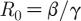 is greater than 1 (Anderson & May 1992; Keeling & Rohani 2007).

### 2.2 Stochastic behaviour and the diffusion approximation

The above equations are however deterministic and deal only with the mean dynamics, and therefore do not capture the variability expected from any biological population. To account for this variability the dynamics are generally made stochastic, such that the population is integer-based and events occur at probabilistic rates. For the SIS equations, the stochastic counterpart is a Markovian process (future dynamics depend only on the current state) and hence a rich mathematical theory can be brought into play. Borrowing from the notation of pairwise models (Keeling *et al*. 1997), we define [*S*](=*NS*) and [*I*](=*NI*) to be the *number* of susceptible and infectious individuals in the population. For a population of size *N*, we can now express the Markovian SIS system by defining the rate of transition between states (*q*(*a*,*b*) is the rate of transition from state *a* to state *b*),2.2
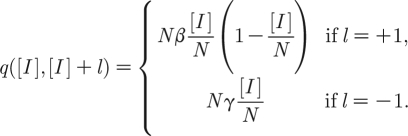

The rates *q* have an obvious matrix formulation, and for this one-dimensional case Kolmogorov forward equations can easily be formulated and solved numerically to give the probability of being in any given state (Keeling & Ross 2008). However, such numerical methods rapidly fail when dealing with very large population sizes or higher dimensional models—we therefore seek more robust analytical methods.

An extremely effective approach for studying stochastic dynamics, particularly when dealing with large population sizes, is to use diffusion approximations. Such processes arise naturally in the framework of continuous-time Markov chains and provide a mathematically rigorous framework, which establishes conditions for convergence in the large population size limit (Kurtz 1970, 1971). Essentially, this framework allows us to replace the integer-based stochastic dynamics for the number of individuals by a stochastic (Gaussian) diffusion process in continuous space modelling the proportions of each state in the population. Moreover, exact analytic results can be formulated to describe the time evolution of variances and covariances within the system. Such models have been used to great effect in ecology (Ross 2006) and epidemiology (Clancy *et al*. 2001).

Given that for a fixed *N* (constant population size) the rates in equation (2.2) can be expressed solely in terms of proportions (i.e. 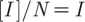), then the (finite-state) Markovian SIS model is a *density-dependent* process and satisfies the criteria for convergence to a Gaussian diffusion process (Kurtz 1970, 1971). For large population sizes this essentially has two main implications: firstly, stochasticity can be captured by including appropriately scaled Gaussian noise to the dynamics and, secondly, the equilibrium dynamics converge to a Gaussian distribution whose variance can be calculated. More specifically, for the SIS model, the first implication means that the Markovian system can be accurately captured with a stochastic ODE model2.3
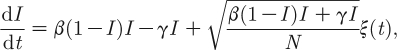

where *ξ* is the Gaussian white noise process with mean 0 and variance 1 (theorems 3.1 and 3.5, Kurtz 1971). Equation (2.3) effectively approximates the integer-based dynamics of equation (2.2) with a continuum-based model; in what follows we refer to equation (2.3) as the stochastic mean-field model (although readers may also prefer to consider this as the diffusion approximation to the stochastic mean-field SIS model) and equation (2.2) as the continuous-time Markov chain of the mean-field model.

The result of the second implication comes directly from the recognition that assuming the process is in equilibrium (that is assuming endemicity has been reached) we have an OU process, and thus the distribution around the equilibrium converges to a Gaussian with variance (Grimmett & Stirzaker 2001)2.4
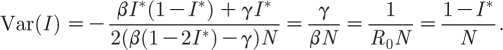

When considering the number of infectious individuals, [*I*], the variance scales to 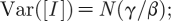 the fact that the number of infected individuals and the associated variance both scale linearly with population size (for large *N*) agrees with a range of simulation results and biological findings (Taylor 1961; Keeling 2000*a*). The above calculations follow the more general methodology given in appendix B. We note that, for such processes and in the large population size limit, the mean of the stochastic process and the deterministic equilibrium are equal.

### 2.3 Pairwise approximations

Pairwise approximation models offer a relatively low-dimensional and parsimonious means of extending standard ODE disease models to incorporate network structure (Keeling 1999; Keeling & Eames 2005). For sexually transmitted infections where the transmission network of sexual contacts generally has limited clustering, the pairwise approximation has been found to produce results in good agreement with network-based simulations, although due to its deterministic nature the pairwise equations are unable to explain the observed variability (Eames & Keeling 2002). Although more complex versions of the pairwise equations have been developed to capture the observed heterogeneity in the number of sexual partners (Eames & Keeling 2002), for simplicity in this paper we deal with the pairwise approximation to a regular network (Caley tree) where every individual has exactly *n* contacts (Keeling 1999). We stress that this is an approximation to the observed highly heterogeneous nature of sexual contact networks, but it allows us to develop a relatively low-dimensional description of the infectious dynamics as individuals are homogeneous.

Using the notation developed by Rand and co-workers (Keeling *et al*. 1997) and described in detail elsewhere (Keeling 1999), we find that, for an SIS-type infection, the number of infectious individuals is determined by2.5
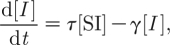

where [*I*] is the number of infectious individuals in the population and [SI] is the number of susceptible–infectious pairs—noting that pairs are counted in both directions and that [SI]=[IS]. In this equation, the transmission occurs at a rate *τ* across any contact between an infectious individual and a susceptible individual, hence including the network structure; moreover, equation (2.5) is an exact description of the deterministic dynamics if we know the number of *S*–*I* pairs. One approach is to approximate the number of *S*–*I* pairs from the number of susceptible and infectious individuals in the population assuming that the constituents of the pair are uncorrelated ([SI]≈[*S*].[*I*]/*N*); this is the random-mixing assumption and leads to the basic SIS model (equation (2.1)). The alternative approach is to formulate equations for the pairs [SI] and [II], which leads to the following coupled ODEs:2.6
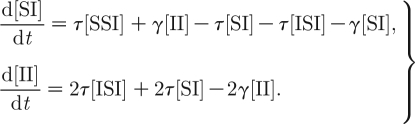

These equations are based on the rates at which particular pair types are created or destroyed; for example, the first term in the first equation relates to the creation of an *S*–*I* pair from an *S*–*S* pair which is part of an *S*–*S*–*I* triple. (A more complete description of this type of formulation is given in Keeling (1999).) We note that again these pairwise equations offer an exact description of the SIS epidemic process on a network, although the formulae require knowledge of the number of triples (e.g. [SSI]) in the population. Using the now standard approach (Keeling 1999), we approximate the number of triples in terms of the number of pairs, assuming the network is a regular random graph—everyone has *n* contacts and these are chosen at random from the population,2.7
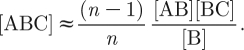

Essentially, this approximation considers a triple to be composed of two pairs that share the central individual but are otherwise independent.

The standard deterministic pairwise model (equation (2.6)) deals with numbers of pairs within the population. To make the scaling within our formalism more precise, we define two new variables, *u* and *v*, which lie between 0 and 1, and which measure the proportion of pairs of type *S*–*I* and *I*–*I,* respectively (*u*=[SI]/(*nN*), *v*=[*II*]/(*nN*)⇒*u*+*v*=*I*). Hence, using the triple approximation (equation (2.7)), the pairwise SIS model can be written in full as,2.8
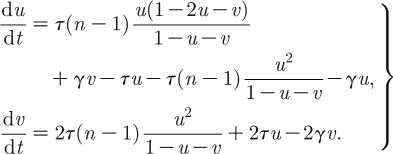

The aim of this paper is now to capture the variability about the solution of equations (2.6) or (2.8) using coupled stochastic ODEs (i.e. a diffusion approximation), and in particular to extract the variance–covariance matrix for this system. Both of these operations can be achieved using standard approaches given that the stochastic formulation of (2.6), combined with (2.7), defines a density-dependent process and satisfies the criteria for convergence to a Gaussian distribution (Kurtz 1970, 1971).

From equation (2.8) it is relatively easy to determine the equilibrium (endemic level of infection)
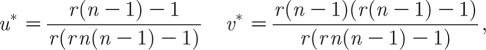

where *r*=*τ*/*γ* must be greater than 1/(*n*−1) and *n*≥2, otherwise the disease always dies out. We therefore see that *r*(*n*−1) has a close analogy to *R*_0_ in standard (non-network) disease models.

In the calculations that follow, in order to achieve a fair comparison between the mean-field SIS model (equation (2.1)) and our pairwise model (equation (2.6) or (2.8)), we insist that both models have the same proportion of infectious individuals at equilibrium
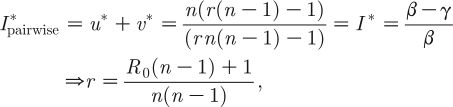

which, as expected, provides agreement at the threshold for successful invasion (*R*_0_=1⇒*r*(*n*−1)=1).

## 3. Stochastic ODEs for the SIS pairwise model

Conversion of the integer-based stochastic pairwise model to its stochastic diffusion approximation should in principle be relatively straightforward, adding a scaled Gaussian noise term for each event that can occur. However, great care is needed in determining precisely the nature of each possible event. For SIS-type disease dynamics only two processes occur—infection and recovery. In the mean-field stochastic model each of these processes is associated with a single event, changing the number of infectious and susceptible individuals by one. However, for a pairwise-based approach, it becomes necessary to consider the entire neighbourhood surrounding the affected individual, this is because infection and recovery can cause a variety of changes to the number of pairs dependent on the constituents of the neighbourhood. For example, consider an infectious individual whose neighbourhood consists of *m* other infected individuals and hence *n*−*m* susceptibles; if this central infectious individual recovers then *m* II pairs and (*n*−*m*) SI pairs are destroyed, but *m* SI pairs and (*n*−*m*) SS pairs are created. From this argument it is clear that we must consider as separate events each process and each neighbourhood configuration. This is a considerable conceptual change. Previously, the pairwise model (as the name suggests) focused on the dynamics of pairs of connected individuals that often necessitated calculation of connected triples but higher order neighbourhood configurations could be ignored; however, the stochastic version of this model while still focusing on the dynamics of pairs requires the consideration of the entire neighbourhood of an individual. This change of perspective may make it easier to include the effects of heterogeneities and local clustering within the network structure. Under this reformulation, the standard pairwise equations (2.6) become:3.1
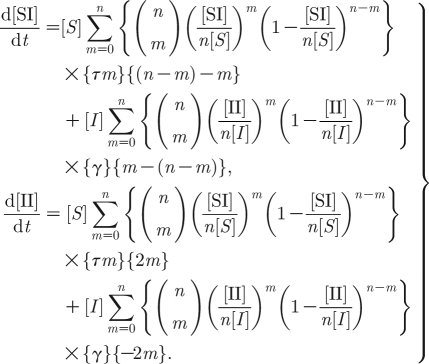

The three elements in braces for each term refer to: the probability of having *m* infectious individuals around the central node (assuming pairwise correlation but uncorrelated triples); the rate at which the event occurs given the neighbourhood; and the change to the number of pairs given the neighbourhood. This new formulation (equation (3.1)) essentially operates at the scale of neighbourhoods surrounding central individuals, but uses the pairwise correlation information to construct these in a weighted manner, although correlations between neighbours and within triples are ignored. We note that, as expected, when the sums in equation (3.1) are evaluated we regain equation (2.6).

The formulation of a stochastic ODE approximation accounting for the variation about equations (3.1) proceeds as before, with each event (corresponding to each neighbourhood configuration and each process) associated with an additional noise term. The full set of stochastic ODEs is shown in appendix A, from which we observe that we can either construct the stochastic ODEs by including one scaled noise term for each event (Keeling & Rohani 2007) or we can use the methods developed by Kurtz (1970, 1971) to construct a 2×2 covariance noise matrix. These two approaches agree due to the independence of the noise terms that arise from considering events rather than processes. An example of aggregated results from numerically integrating equations (A 1) or (A 2) is given in figure 1; note that as expected the mean of the two stochastic ODEs (equations (2.3) and (A 1) or (A 2)) agree and is equal to the deterministic mean. From figure 1 *a*, we observe comparatively close agreement between the stochastic pairwise and stochastic mean-field results in terms of the variance in the total number of infectious individuals, [*I*]—noting that the transmission parameter of the stochastic model has been rescaled to achieve agreement in the deterministic equilibrium. The pairwise model has a slightly higher variance and this is found to be a consistent feature of all parameter regimes tested. Figure 1 *b*,*c* provides more insight into the constituent pairwise components of this variation. Firstly, we note the negative correlation between the proportion of *S*–*I* pairs (*x*-axis) and the proportion of *I*–*I* pairs or the proportion of infectious individuals (*y*-axis). In addition, for these parameters, the variation associated with *S*–*I* pairs is far less than the variation in *I*–*I* pairs, and so it is this latter variation that drives the overall variance in the level of infection.

**Figure 1:**
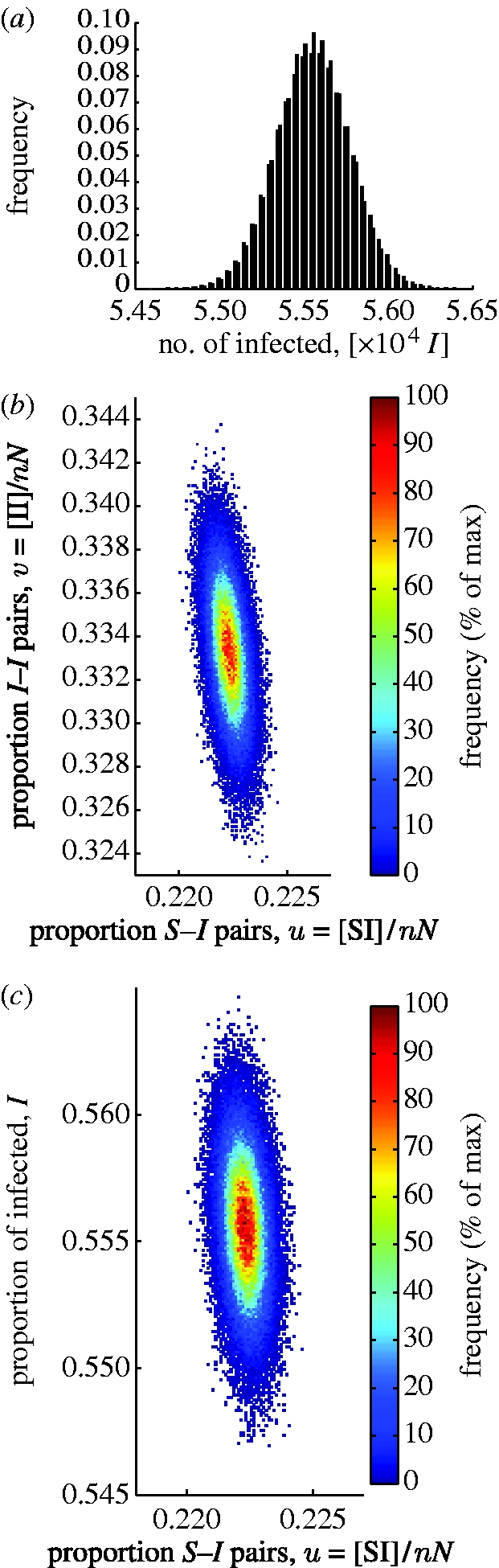
Distributions from the stochastic ODEs (equations (2.3), (A 1) and (A 2)). (*a*) compares the distribution of the number of infectious individuals from stochastic pairwise (black bars) and mean-field (white bars) versions of the SIS model; the pairwise model can be seen to give rise to a slightly larger variation. (*b*) and (*c*) are from the stochastic pairwise model only, and consider the covariance between the proportion of *S*–*I* pairs and either the proportion of *I*–*I* pairs or the proportion of the population that is infectious. Here, proportions are used such that axes can be plotted on equal scales, and we plot the frequency as a percentage of the maximum for ease of comprehension. (*τ*=0.05, *γ*=0.01, *n*=5, *N*=100 000⇒*r*=0.5, *R*_0_=2.25, *β*=0.225, *I* ^*^=5/9. All results are from 100 000 time steps and ignore the initial transient dynamics.)

While equations (A 1) and (A 2) in appendix A provide a means of numerically integrating the stochastic dynamics of the pairwise equations, they do not provide an easy analytical comparison with the results derived for the stochastic SIS model (equation (2.4)). We therefore use equation (2.8) together with the standard results for OU processes (Grimmett & Stirzaker 2001) to derive the distributions around the mean equilibrium value for large population sizes. The full covariance matrix for the variables *u* and *v* is cumbersome and consequently not overly informative (see appendix B). We therefore focus primarily on the variance in the proportion of the population that are infectious, and undertake numerical comparisons between the stochastic mean-field SIS model and the stochastic pairwise equations to inform about the interactions between noise and network processes. The variance from the pairwise model is given by:
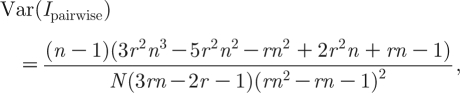

whereas, for the mean-field model (assuming the same equilibrium value), we have
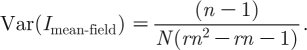

Examining the ratio of the two variances we find that:3.2
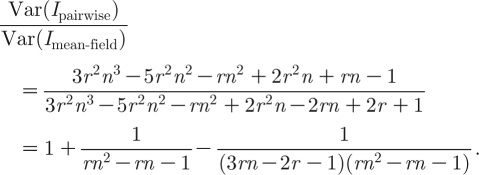

Hence, the stochastic pairwise model always has a greater variance than the stochastic mean-field model, but the ratio drops to 1 as either *r*, *n* or *R*_0_ becomes large. In part this is to be expected as the mean-field model can be considered as the limiting case of the pairwise model as *n* becomes large, and hence we would expect agreement. When *r* is relatively large, then both models predict that most of the population is infected leading to little spatial structure within the network (as captured by the pairwise equations) and so the stochastic mean-field model is an accurate approximation to the full dynamics.

Figure 2 shows the bivariate normal distribution for various combinations of the key parameters, which should be compared with the numerical results from the stochastic version of the pairwise and mean-field models shown in figure 1. These results confirm our earlier findings and validate the analytical calculations given in the appendices. The close agreement between the stochastic mean-field (thick black line) and stochastic pairwise (shaded area) models, in terms of variation in the number of infectious individuals, is particularly striking. For the parameters used in figures 1 and 2, table 1 details the numerical values of various quantities of interest for both the stochastic pairwise model and the mean-field equivalent. For completeness results from a full Monte Carlo network simulation are also given in table 1, allowing us to assess the accuracy of both models; we find that in general the pairwise model performs well with errors being attributable to slight differences in rates of convergence to the equilibrium caused by ignoring triple correlations. For this particular choice of parameters, we observe some significant differences between the stochastic pairwise model and the stochastic mean-field model even though they are parametrized to generate the same equilibrium level of infection. In particular, the deviation between the stochastic mean-field and the full Monte Carlo network simulation is generally far greater than the deviation between the pairwise model and the network simulation; most strikingly the variance in *S*–*I* pairs is poorly captured by the stochastic mean-field approximation.

**Figure 2:**
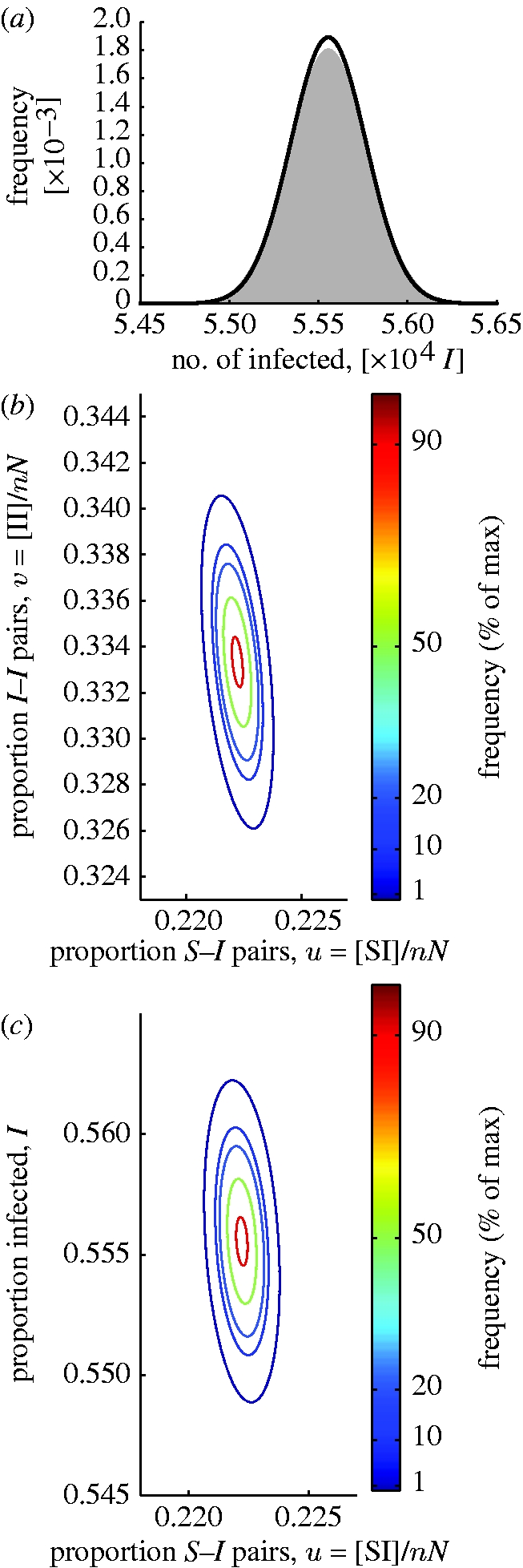
Distributions from the bivariate normal distributions predicted from the dynamics of the stochastic SIS pairwise (black bars) and mean-field (white bars) models (equation (2.4) and appendix B). Graphs and parameters are exactly as in figure 1 for ease of comparison. (*b*) and (*c*) show the 1, 10, 20, 50 and 90% contours.

**Table 1:** Comparison of key distributional values for a full Monte Carlo (continuous-time Markov chain) network simulation, the stochastic pairwise and the stochastic mean-field models. (The network simulation uses full asynchronous updating of the disease status of each node (Gillespie 1977; Keeling & Eames 2005; Keeling & Rohani 2007), a regular network where each node has five contacts. The network simulation and pairwise model share the same individual-level parameters (*τ*=0.05, *γ*=0.1, *n*=5, *N*=100 000), while the mean-field model is again parametrized to have the same equilibrium level of infection as the pairwise model. The pairwise results come from the covariance matrix calculated in appendix B. The stochastic mean-field model is parametrized to have the same equilibrium level of infection, while the pairwise values (for the mean-field model) are calculated making the mean-field assumption of ignoring spatial correlation within the pair. The values in brackets show the percentage deviation of the stochastic pairwise and mean-field results away from the simulated values; clearly the pairwise model consistently performs better.)

quantity	Monte Carlo network simulation	stochastic pairwise (% deviation)	stochastic mean-field (% deviation)
mean number infected, [*I*]^*^	5.4993×10^4^	5.5556×10^4^ (1.0%)	5.5556×10^4^ (1.0%)
mean number *I*–*I* pairs, [SI]^*^	1.0997×10^5^	1.1111×10^5^ (1.0%)	1.2346×10^5^ (12.3%)
mean number *I*–*I* pairs, [II]^*^	1.6500×10^5^	1.6667×10^5^ (1.0%)	1.5432×10^5^ (6.5%)
variance in [*I*]	5.0057×10^4^	4.8485×10^4^ (3.1%)	4.4444×10^4^ (11.2%)
variance in [SI]	7.1892×10^4^	6.6667×10^4^ (7.3%)	1.3727×10^4^ (80.9%)
variance in [II]	1.4262×10^6^	1.4202×10^6^ (0.4%)	1.3718×10^6^ (3.8%)
covariance between [*I*] and [SI]	−1.0272×10^4^	−1.4141×10^4^ (37.7%)	−2.4691×10^4^ (140.4%)

Figure 3 extends the above analysis across a range of parameter values; in particular, as the number of neighbours *n* and the equilibrium (endemic) level of infection *I* ^*^ varies. From all these figures it is clear that the maximum deviation from the stochastic mean-field results (shown as a thick black line) occurs when the number of contacts, *n*, is small and when the equilibrium level of infection is low. It is also apparent that the convergence to mean-field happens rapidly with increasing *n*. As expected, the ratio of the pairwise variance in the level of infection to the mean-field variance shows that the pairwise model always experiences greater variability (figure 3 *a*)—with the ratio tending to 5/3 when *n*=2 and as *I* ^*^→0. For each of the figure 3 *b–d*, the stochastic mean-field results (shown as thick black lines) that are equivalent to the large *n* limit of the stochastic pairwise model can be calculated in terms of the equilibrium level of infection and the stochastic mean-field variance:
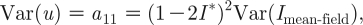


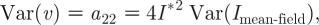


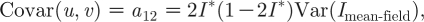

where the variance in the level of infection Var(*I*_mean-field_)=(1−*I* ^*^)/*N*. Examining figure 3 *b*,*c*, we observe that, whenever the equilibrium level of infection is relatively high (greater than 0.2) or when *n* is very small, the variance in *v* (associated with *I*–*I* pairs) dominates the variance in *u* (associated with *S*–*I* pairs), and hence contributes the most to the variance in the level of infection; this is the case for the parameters in figures 1 and 2. Finally, in figure 3 *d*, we consider the covariance between *u* and *v* and note that, although the precise value of this covariance changes with *n*, the point where the covariance is 0 always occurs at *I* ^*^=1/2.

**Figure 3:**
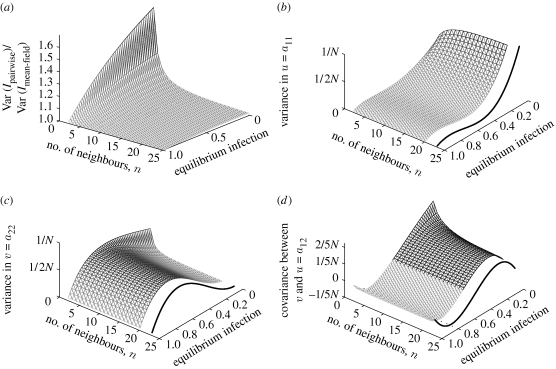
Comparison of stochastic pairwise and mean-field results using the predicted bivariate normal distributions. All graphs are plotted for a range of equilibrium infection levels (0<*I* ^*^<1) and for the various numbers of contacts (2≤*n*≤20); we equate equilibrium levels rather than transmission rates as this gives a more natural interpolation between pairwise and mean-field results. In (*b–d*), the thick black line gives the large *n* limit of the pairwise model calculated from the predicted mean-field variance. (*a*) shows the relative variance in the level of infection for the stochastic pairwise model compared with the stochastic mean-field, Var(*I*_pairwise_)/Var(*I*_mean-field_) (equation (3.1)); (*b*–*d*) show the variance in *u*, the variance in *v* and the covariance between them, respectively.

## 4. Variance during early growth

The above comparisons have all used methodologies developed for the long-term stochastic dynamics around the equilibrium. However, comparable, but more complex, formulae exist (appendix C) that deal with variation about temporally varying population dynamics. We use this formalism to examine the dynamics following invasion in the particular case where both deterministic and pairwise SIS models predict exponential growth of infection at rate *κ* (e.g. *I*(*t*)=*I*(0)exp(*κt*)). For the model we find that *κ=β*−*γ* and hence *κ* is positive whenever *R*_0_ is greater than 1. For the pairwise model the calculation of early growth rates is more complex; either we can use an eigenvalue approach on the SIS system of equations (2.6) or we can note that [SI] and [II] (or equivalently *u* and *v*) must also grow at rate *κ* and calculate their ratio. In either case, exponential growth occurs only once initial transient dynamics have died away and a consistent spatial structure has developed (setting the ratio of [SI] to [II]; Keeling 1999). We equilibrate the exponential growth rates, rather than simply *R*_0_, for two reasons: firstly, this fitting ensures that (after transients) the two deterministic models have identical early behaviour and, secondly, because it is the early growth rate of an epidemic that is measured in practice.

The results in appendix C therefore provide an analytical understanding of the variation about the expected exponential growth rate of infection once initial transient dynamics have decayed. We first note that both stochastic mean-field and stochastic pairwise models predict that the variance grows such as exp(2*κt*); or equivalently that the standard deviation (square root of the variance) grows at the same rate as infection. A comparison between the relative variances predicted by stochastic mean-field and stochastic pairwise models is shown in figure 4; this should be compared with figure 3 *a*, which shows similar relative variances at equilibrium. Three main conclusions can be drawn. Firstly, for the majority of parameter space the variance predicted by the stochastic pairwise model is less than that predicted by the mean-field equivalent; the only exception occurs when *n*=2 and the growth *κ* is large. The anomalous behaviour occurs because when *n*=2 the network is reduced to a simple line; this places strong constraints on the early growth of infection such that 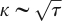, whereas when *n*>2 we find that *κ*∼*τ* (appendix C). Secondly, for each value of *n* there is a growth rate *κ*_min_ (close to the recovery rate, *γ*) that minimizes the relative variance in the pairwise model. Finally, because we have forced both models to agree on the population-level dynamics (by fixing the early growth rate) we again find that the predictions of stochastic mean-field and pairwise models are in far closer agreement than if we had simply matched the individual-level behaviour.

**Figure 4:**
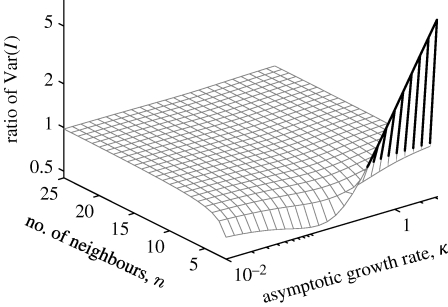
Comparison of stochastic pairwise and mean-field results using the predicted bivariate normal distributions for the stochastic dynamics during the early exponential growth phase following invasion into a totally susceptible population. In particular, we compare the variance in the level of infection (Var(*I*_pairwise_(*t*))/Var(*I*_mean-field_(*t*)); appendix C), when both deterministic and pairwise models are parametrized to have a growth rate *κ*. Lines are pale grey when the ratio is less than 1 and black when the ratio is more than 1. (Here, we set *γ*=0.1, and note that the population size *N* is irrelevant as long as it is assumed to be large.)

## 5. Discussion

Three main conclusions can be drawn from this work. The first is that, with careful separation of all possible events (as opposed to processes), it is relatively straightforward to consider how stochasticity influences the dynamics of pairwise models and hence how stochasticity and local spatial structure interact. The separation of events effectively leads us to work with neighbourhoods of individuals (rather than connected pairs) and use the pairwise correlations to define the distribution of neighbourhood types—assuming neighbours are independent leading to a multinomial distribution of neighbourhoods. This is a considerable conceptual change from the standard pairwise models that deal solely with contacts; the ability to deal with entire neighbourhoods opens up the possibility to consider far more complex network structures centred on individuals.

The second, more striking, conclusion is that for the vast majority of parameter space the stochastic pairwise model and (a suitably parametrized) stochastic mean-field model have very similar variation in the total level of infection close to the endemic equilibrium. We now speculate as to why this occurs. Consider the deterministic mean-field and pairwise equations, formulated for the number of infectious individuals,
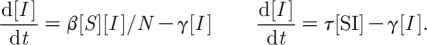

When these two models are parametrized to have equal levels of infection at equilibrium, it is clear that *β*[*S*][*I*]/*N*=*τ*[SI]. Given that the noise associated with the level of infection (e.g. equation (2.3)) is defined in terms of the rates of change, the instantaneous noise experienced by the stochastic mean-field and pairwise model *at equilibrium* must be identical. Therefore, the only source of variation between the two models is the deviation of *τ*[SI] away from *β*[*S*][*I*]/*N*. Examining the correlation between [SI] and [*I*], which is captured by 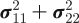 (appendix B), shows only a relatively slight deviation between the stochastic pairwise model and the mean-field limit. Additional deviation may be caused by the noise associated with the [SI] term, whereas in the mean-field limit this term is proportional to the variation in the level of infection; however, in the large population size limit that we are considering here such effects are small. We therefore conclude that, except when both the number of contacts and the equilibrium level of infection are small, the act of forcing the two deterministic models to have equal equilibria leads to equal rates of infection and recovery at the equilibrium, which in turn leads to comparable levels of noise being experienced by the stochastic version of these models. Of course, the pairwise model also experiences noise in the correlation between connected individuals, but the impact of this on the dynamics is minimal when the population size is large.

Given this similarity in the level of variation predicted for stochastic pairwise and stochastic mean-field models, the usefulness of these results (and network/pairwise models in general) may be questioned; however, we believe that these models are both useful and informative. A wide body of literature has demonstrated that network models can generate very different dynamics compared with the mean-field equivalent (Keeling & Eames 2005; Boccaletti *et al*. 2006); however, stochastic differences have rarely been elucidated. The similarity in stochastic variation observed here is both non-intuitive and critically dependent on our careful matching of the mean dynamics. We therefore feel that the development of the stochastic pairwise methodology is both informative and likely to have significant applied impact when used to consider more complex network constructions that include heterogeneity and clustering.

The final conclusion concerns the early dynamics following invasion. Here, except for the anomalous case when *n*=2, the stochastic pairwise model is predicted to have a lower variance than its mean-field counterpart; with the relative difference being greatest when the number of contacts *n* is small and the growth rate *κ* is comparable with the recovery rate *γ*. As with the variance around the equilibrium, the relative differences between mean-field and pairwise predictions are not huge and generally are within a factor of two—the same reasoning as above still holds given that we are now forcing both models to have the same exponential growth rate. We speculate that spatial structure has a stabilizing effect (lower variance) during the exponential growth phase because there is negative feedback via the spatial correlation captured by *S*–*I* pairs; an infectious individual who by chance infects more of its contacts than expected depletes its local environment of susceptibles and therefore its subsequent chance of generating further cases is reduced. Simply put, for the regular network considered here, each infectious individual is limited to cause at most *n* secondary cases which in turn limit the noise associated with each generation compared with the mean-field approximation.

In summary, if we assume that network and mean-field models are parametrized to match the same epidemic growth rate data, then the lower variation predicted in the stochastic version of the pairwise model (compared with the stochastic mean-field model) is likely to lead to a lower level of stochastic extinction in the pairwise model. We therefore postulate that stochastic network-based models should be easier to invade (be less prone to early stochastic extinction) than their stochastic mean-field counterparts, although their latter dynamics are subject to larger fluctuations.

In this paper, we have concentrated on developing a standard formulation within which noise can be incorporated into the pairwise modelling approach in a manner that scales appropriately with the rates at which events occur. We have taken the simplest of epidemiological models—the SIS model and the simplest network structure—a random network in which each individual has exactly *n* contacts. Many subsequent improvements to this general approach remain to be made. The methodology could equally be applied to the alternative pairwise formulation of Boots & Sasaki (1999, 2002) to provide insights into noise on lattice-based models. While the SIS paradigm is a good approximation for sexually transmitted infections and many host–parasite diseases, other epidemiological models could be considered, such as SIR or SEIR type dynamics; however, given the similarity in the infection equation for all these models we again expect stochastic pairwise and suitably parametrized stochastic mean-field models to experience relatively similar levels of variability. Of more importance is heterogeneity and clustering within the network structure—we speculate that having some highly connected (high-risk) individuals within the population or having the population composed of tightly interconnected groups is likely to impact substantially on the level of noise experienced. Bringing together the existing formalism to deal with heterogeneous or clustered pairwise models (Keeling 1999; Eames & Keeling 2002) with the method of including noise outlined in this paper is clearly possible but remains a challenge for the future.

## Appendix A. Stochastic ODE model for the pairwise equations

A1

where *ξ*_*m*_ and *ζ*_*m*_ are independent Gaussian white noise processes (mean 0 and variance 1) associated with transmission and recovery, respectively.

This can also be expressed with the formulation by Kurtz (1970), which generates a somewhat simpler set of equations:

A2

where *ξ* and *ζ* are independent Gaussian white noise processes (mean 0 and variance 1) and the matrix ***A*** is defined as the square root of the symmetric matrix ***H***, whose terms are given by the appropriate sums of rates and changes to [SI] and [II],

A3

## Appendix B. Covariance matrix

From standard theory for density-dependent processes and convergence to an OU process, we find that the covariance matrix ***σ*** ^2^ satisfies

where

B1

where *f*_*e*_ is the rate at which event *e* occurs; *F* is the total rate of change to each variable; and local drift matrix ***B*** is the Jacobian of *F* evaluated at the endemic fixed point. Finally, the local covariance matrix ***G*** takes into account the changes to the two variables due to the total set of events, and measures absolute rates of change which in turn are related to the stochastic noise. (Δ*u*_*e*_ is defined as the total change to *u* due to event *e*.) We note that ***G*** is the equivalent of ***H*** given in appendix A, but defined in terms of *u* and *v* rather than [SI] and [II]. Using the above notation and operating in terms of *u* and *v*, the full stochastic equations (A 1) given in appendix A reduce to

B2

The matrices ***B*** and ***G*** can be evaluated by summing over all possible events and using the associated rates and changes to the two variables. From these we find that the covariance matrix ***σ*** ^2^ is symmetric with components (***σ*** ^2^)_*ij*_. Where

where the term *nN* in the denominator is assumed to be large and comes from the fact that we are dealing with the proportion of all pair types. Although involved, these terms can easily be evaluated numerically and readily inform about the stochastic dynamics. In particular,

## Appendix C. Early growth and the associated covariance matrix

We are now concerned with expanding the methodology in appendix B to deal with the situation where the population is not at equilibrium. The work by Kurtz (1970, 1971) again provides the appropriate theory; the time-varying covariance matrix ***σ*** ^2^(*t*) can be expressed as

C1

where

where the matrices ***B*** and ***G*** are defined as in appendix B, except that they are evaluated at the time-varying trajectory of the population.

We now consider the particular case of the early exponential growth of an epidemic in a naive population (*I*(*t*)≪1), and will constrain the parameters such that the deterministic and pairwise model have the same exponential growth rate, *I*(*t*)∼*I*(0)exp(*κt*). Under these assumptions, we find that ***B***(*t*) is constant, while ***G***(*t*) can be expressed as —where  is constant. Integrating equation (C 1) by parts yields the following identify:

C2

C3

where the long-term behaviour is derived from the fact that exp(***B***) determines the dynamics of the linear system.

From equations (C 2) and (C 3), we find for the one-dimensional stochastic model that

For the pairwise models, the expressions are obviously more complex. Linearizing equation (2.8) allows us to determine simple relationships for the early dynamics

while the matrices are given by

where  and

where *q*=*v*(*t*)/*I*(*t*)→2(*κ*+γ)/(*nκ*+2*nγ*).

From these expressions and using equation (C 3) we can calculate the time-varying covariance matrix for the pairwise model, noting that this formula holds only when the exponential growth phase has been proceeding for some time, such that the ratio of *u* and *v* has converged to a quasi-equilibrium and the exponential of ***B*** has also converged to its asymptotic behaviour. The rate of this convergence is determined by the dominance of the first (positive) eigenvalue compared with second (negative) eigenvalue of the system.
